# Ab Initio Calculation of Coupling-Constant Averaged Exchange–Correlation Holes for Spherically Symmetric Atoms

**DOI:** 10.1021/acs.jpca.4c02717

**Published:** 2024-09-23

**Authors:** Lin Hou, Tom J. P. Irons, Yanyong Wang, James W. Furness, Andrew M. Wibowo-Teale, Jianwei Sun

**Affiliations:** † Department of Physics and Engineering Physics, Tulane University, New Orleans, Louisiana 70118, United States; ‡ School of Chemistry, University of Nottingham, University Park, Nottingham NG7 2RD, United Kingdom

## Abstract

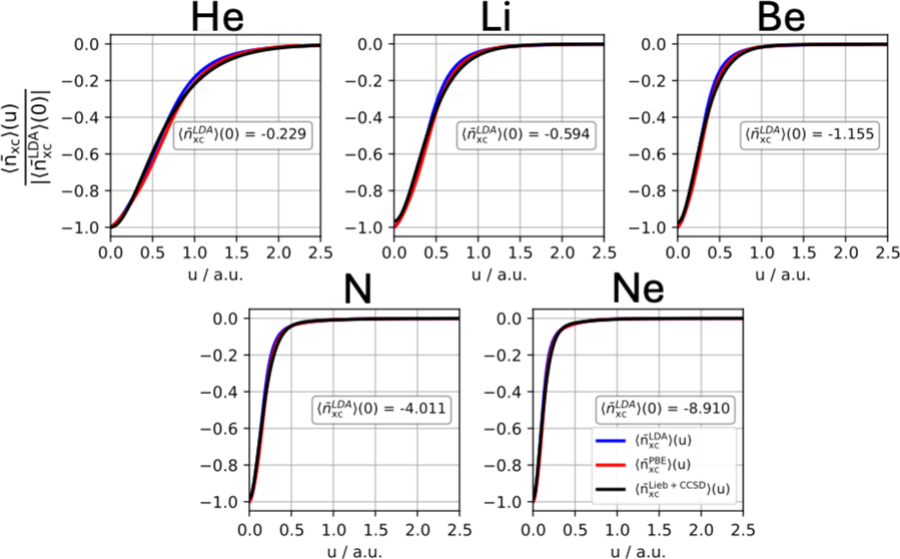

Accurate approximation of the exchange–correlation (XC) energy in density functional theory (DFT) calculations is essential for reliably modeling electronic systems. Many such approximations are developed from models of the XC hole; accurate reference XC holes for real electronic systems are crucial for evaluating the accuracy of these models however the availability of reliable reference data is limited to a few systems. In this study, we employ the Lieb optimization with a coupled cluster singles and doubles (CCSD) reference to construct accurate coupling-constant averaged XC holes, resolved into individual exchange and correlation components, for five spherically symmetric atoms: He, Li, Be, N, and Ne. Alongside providing a new set of reference data for the construction and evaluation of model XC holes, we compare our data against the exchange and correlation hole models of the established local density approximation (LDA) and Perdew–Burke–Ernzerhof (PBE) density functional approximations. Our analysis confirms the established rationalization for the limitations of LDA and the improvement observed with PBE in terms of the hole depth and its long-range decay, which is demonstrated in real-space for this series of spherically symmetric atoms.

## Introduction

Density functional theory (DFT) has become the most widely used electronic structure method on account of the high accuracy that can be achieved with relatively low computational scaling in the study of many-body systems. DFT is now the mainstay of electronic structure modeling across a range of areas including condensed matter physics, quantum chemistry and materials science. Through determining the ground-state electron density directly, the construction of a many-body wave function can be avoided, while allowing the ground-state energy and important chemical and physical properties of the system to be evaluated.^1^

Most practical implementations follow the Kohn–Sham formulation of DFT (KS DFT), in which the density is represented by a set of one-electron orbitals that form a single determinant wave function.^2^ Besides being computationally convenient, this approach enables the largest contributions to the energy to be evaluated exactly, requiring approximation of the remaining exchange–correlation (XC) component, responsible for many-electron effects. It is therefore the quality of the XC density functional approximation (DFA) that determines the accuracy of the energy and other ground-state properties predicted by KS DFT.^3^

One approach to the development of XC DFAs has been by modeling coupling-constant averaged XC hole at position **r** + **u** around an electron of spin σ_1_ at position **r**, which is denoted by *n̅*_xc_^σ_1_σ_2_^(**r**,**r** + **u**). The XC energy may be evaluated from this quantity as^4−6^
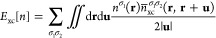
1in which *n*^σ_1_^ represents the σ_1_-spin density. This may be resolved into separate exchange and correlation holes, denoted *n*_x_^σ_1_^ and *n̅*_*c*_^σ_1_σ_2_^ respectively, as
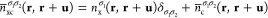
2where δ_σ_1_σ_2__ is the Kronecker delta, which ensures only parallel spin contributions to the exchange energy. The accurate calculation of the XC energy therefore relies on the quality of the XC hole model, the assessment of which is instructive in understanding the limitations of XC DFAs when applied to real systems and developing more reliable models.^7^ However, the tendency to directly model the XC energy density in the development of widely used DFAs often overlooks the significance of XC holes, with the result that studies focusing on XC holes have been relatively limited.

Notably many successful DFAs, in particular nonempirical models, were based on insights of the XC hole. Recently, the significance of XC holes is being recognized again in new DFA developments.^8^ Among the earliest DFAs is the local density approximation (LDA) introduced in 1965 by Kohn and Sham^2^ and derived from the uniform electron gas. The success of LDA for real systems was not elucidated until subsequent studies, such as those by Langreth and Perdew^6^ and Gunnarsson and Lundqvist,^4^ which demonstrated that LDA satisfied exact constraints on the exchange–correlation hole, such as the sum rules. Early nonempirical generalized gradient approximations (GGAs), such as the Perdew–Wang 91 (PW91) model,^9^ were developed from the second-order gradient expansion for the exchange–correlation hole, with real-space cutoffs introduced to maintain the exact hole constraints. The Perdew–Burke–Ernzerhof (PBE) density functional^10^ was constructed through two methodologies: one based on fulfilling exact constraints and appropriate norms for the XC holes,^11^ and the other on satisfying exact constraints and appropriate norms for the integrated energy.^10^

The XC hole models for LDA exchange and correlation,^12,13^ in addition to the PBE exchange hole,^12^ were smoothly reverse-engineered from their respective density functionals to recover the corresponding energies, with exact constraints imposed on the model XC holes. The PBE correlation hole^11^ however was not reverse-engineered but was instead constructed first from sharp real-space cutoffs of the second-order gradient expansion, and its integrated energy was shown to agree approximately with that from exact constraints on the energy. Some artifacts resulting from these sharp cutoffs can appear even in the system and spherically averaged correlation hole.

The reverse-engineered PBE exchange hole model plays an essential role in the development of range-separated hybrid density functionals, which combine semilocal density functionals with nonlocal exact exchange through separating the electron–electron interaction into short-range and long-range components. For example, in the long-range corrected ωPBE (LC-ωPBE) hybrid density functional,^14^ the short-range exchange component is derived by applying the attenuated Coulomb operator on the PBE exchange hole model and then integrating over the interelectronic separation. Typically, the correlation hole models for semilocal density functionals, such as the PBE correlation hole model, are not employed in the construction of range-separated hybrid functionals.

The successful nonempirical meta-GGAs, including the Tao–Perdew–Staroverov–Scuseria (TPSS) density functional,^15^ were developed following the second approach used in constructing the PBE functionals; satisfying exact constraints and appropriate norms for the integrated energy. The TPSS exchange and correlation holes were constructed in a similar manner to that in which the PBE exchange and correlation holes were defined.^16^ The understanding of XC hole properties has played a pivotal role in the construction of the strongly constrained and appropriately normed (SCAN) meta-GGA.^17^ The design of SCAN was based on a combination of imposing exact constraints on the functional and fitting to appropriate norms—systems for which a meta-GGA can be exact or highly accurate. These appropriate norms for a meta-GGA are systems in which the exact XC hole is localized near its electron, such as the uniform electron gas and atoms. An additional advantage is that SCAN is not fitted to any bonded system, and makes unbiased but accurate predictions for bonding.

One class of density functional approximation for which correlation hole models are not currently available are the nonlocal correlation functionals designed to capture the effects of van der Waals interactions, such as the Vydrov-Van Voorhis density functional.^18^ The development of correlation hole models for such density functionals would be an intriguing direction for future research.

The benchmarking of model XC holes against accurate reference data remains a challenging yet essential endeavor in the development of DFAs. Numerous studies have explored the exact exchange hole for a small range of systems, including the jellium surface^19^ and the H, He, Li, N and Ne atoms.^5,12,16^ Additionally, investigations have been conducted on the exact correlation hole at full interaction strength (as opposed to the coupling-constant averaged form most appropriate to model in DFT) for Hooke’s atom and the He, Li, Be^2+^, Be and Ne atoms.^20,21^

Evaluating accurate coupling-constant averaged XC holes is not a trivial task; the adiabatic connection (AC) must be constructed between the noninteracting and the physically interacting limits, with the density remaining constant at the physically interacting density throughout. This requires the effective potential to be optimized at each interaction strength such that it yields the physical density, for which the Lieb optimization provides a robust framework.^22,23^ In combination with a coupled cluster singles and doubles (CCSD) reference, the Lieb optimization has been used to study two-electron systems, particularly focusing on the XC energy of the Helium isoelectronic series and H_2_ molecule.^24,25^ More recently, this approach has been employed by the present authors to investigate the influence of the electron–electron cusp on finite basis set calculations of the XC hole using Hooke’s atom as a model.^26^

In the present work, we employ a similar approach to study the XC holes in a set of five spherically symmetric atoms: He, Li, Be, N and Ne. Atoms in this set have between two and ten electrons and this set contains both atoms with closed-shell and open-shell ground-state electronic configurations, allowing the relationship between number of electrons, electronic spin state and XC hole characteristics to be investigated. Moreover, these atoms have been extensively studied in prior research,^20,21^ providing a valuable benchmark for the comparison and validation of the present work. By focusing on these five atoms, our objective is to develop a more detailed understanding of XC holes and their distinctive features in a diverse range of atomic systems.

We further note the greater availability of accurate reference data for XC holes would not just be beneficial for benchmarking model XC holes but would have enormous potential to provide the training data for machine-learned DFT models of the XC hole. The rapid advances in machine-learning and artificial intelligence technology in recent years has led to an enormous growth in the use of such approaches for the development of new models for the XC energy in DFT; see for example refs (27−29) for recent reviews of this progress. Among the most prominent of these advances is the Deep Mind 21 machine-learned functional of Kirkpatrick et al.,^30^ however it was recently shown that even the sophisticated approach taken to construct this model and the broad training set employed was insufficient to ensure a reliable performance when applied to transition metal compounds.^31^ An interesting alternative approach employed by Cuierrier et al.^32^ is the construction of a machine-learned model for the XC hole from the exact conditions which the XC hole is known to satisfy. This approach remains limited by the lack of reference data for the XC hole outside the regions of these exact constraints on which the machine-learning models can be trained, with the greater availability of such data potentially being essential to furthering this approach to machine-learned DFT functional development.

We commence in the Theory section by providing an overview of the theoretical framework for computing the coupling constant-dependent system and spherically averaged XC holes for open-shell systems. The details of the calculations undertaken in this work are then discussed in the Computational Details section. In the Results and Discussion section, we present the coupling-constant averaged XC holes and separate the exchange and correlation holes for these five atoms evaluated using the Lieb optimization with a CCSD reference, examining the convergence of the energy with both basis-set size and interelectronic separation, *u* = |**r**′ – **r**|, over which the holes are integrated. In addition, we study the convergence of the integrals of the exchange and correlation holes with respect to *u* toward the known values of their respective integrals over all space: the *sum rule* criteria for exchange and correlation holes. We apply this analysis to the model exchange and correlation holes of the two most foundational DFAs, namely LDA^12,13^ and PBE,^11,12^ comparing their characteristics with those of the accurate Lieb + CCSD data and rationalize their differences. We conclude our work with a brief summary in the Conclusions section.

## Theory

In KS-DFT, the ground-state energy of a many-electron system in an external potential *v*_ext_(**r**) is obtained by mapping the interacting system of electrons to an auxiliary noninteracting system of electrons with the same density. The problem is then reduced to a set of one-electron equations for the eigenfunctions of the noninteracting Hamiltonian, the solutions to which are a set of orbitals in which the density is represented.^2^ The ground-state energy as a functional of the electron density *n*(**r**) can be resolved into the sum of several contributions as
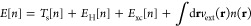
3where *T*_s_ is the noninteracting kinetic energy, which is evaluated exactly using the KS orbitals, and *E*_H_ the classical electrostatic Hartree energy, which is evaluated exactly in terms of the electron density *n*(**r**). The only term in eq 3 which must be approximated is the XC energy *E*_xc_[*n*], which describes all of the many-electron effects in the system.

The KS noninteracting system may be linked to the physically interacting system by continuously varying the strength of the electron–electron interaction between the noninteracting and physically interacting limits, scaling the two-electron operator *V̂*_ee_ by a coupling-constant λ between zero and one. The electronic state evolves through a family of solutions to the λ-interacting Hamiltonian
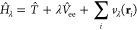
4where *T̂* is the kinetic energy operator and *v*_λ_ a modified external potential, thus establishing an adiabatic connection between the noninteracting and physically interacting systems.^6^ The modified external potential *v*_λ_ is determined for each interaction strength such that the density remains constant at the physical (λ = 1) density for all λ; it reduces to the local KS potential *v*_s_ at λ = 0 and is equal to the physical external potential *v*_ext_ at λ = 1.

Given the normalized ground-state *N*-electron wave function of the λ-interacting system Ψ_λ_, the diagonal of the spin-resolved two-particle density matrix is expressed as^33,34^
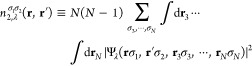
5While the two-particle density cannot be diagonalized by a unitary transformation of the one-electron orbitals,^33^ it may be used to evaluate the expectation value of two-body operators.^7^

The spin-resolved XC hole *n*_xc,λ_^σ_1_σ_2_^(**r**, **r**′) is computed from the spin-resolved two-particle density matrix *n*_2,λ_^σ_1_σ_2_^ and the spin densities *n*^σ^ as
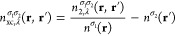
6At λ = 0, the opposite spin component of XC hole *n*_xc,λ = 0_^σσ′^(**r**, **r**′) = 0 while the same spin component reduces to the KS exchange hole
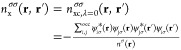
7where ψ_*i*σ_(**r**) are the KS spin–orbitals. Therefore the spin-resolved λ-averaged correlation hole can be defined by
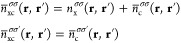
8The XC holes may be spherically averaged by integration over the volume element of the second electron coordinate as
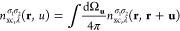
9where **u** = **r**′ – **r**. The system-averaged spin-resolved XC hole is then evaluated by spatially integrating the spherically averaged XC hole with the spin density of the reference electron, then dividing by the number of spin σ_1_ electrons *N*_σ_1__
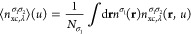
10The spin σ_1_σ_2_ component of the XC energy *E*_xc_^σ_1_σ_2_^ is evaluated by integrating the σ_1_σ_2_ system- and spherically averaged XC hole of eq 10 with respect to the interelectronic distance *u* and multiplying by the number of spin σ_1_ electrons as
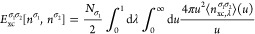
11The total system- and spherically averaged XC hole for an open-shell system is constructed from the sum of the four individual spin terms αα, ββ, αβ, and βα, each evaluated individually according to eq 10, giving the total expression for the system- and spherically averaged XC hole as
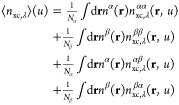
12Similarly, the total XC energy for an open-shell system evaluated in this way is constructed from each of the four components *E*_xc_^αα^, *E*_xc_^ββ^, *E*_xc_^αβ^, and *E*_xc_^βα^ evaluated according to eq 11, to give the total XC energy as
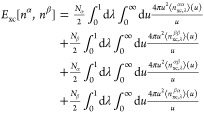
13Using spin-scaling relations, the exchange hole for a spin-unpolarized density *n*_x_[*n*] can be generalized to the spin-polarized density *n*_x_[*n*^σ_1_^, *n*^σ_2_^]
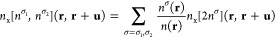
14hence it is only necessary to model the exchange hole as a functional of the total density. Furthermore, the exact system- and spherically averaged exchange and correlation holes satisfy the following sum rules respectively^4,6,7^
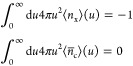
15

The DFT XC energy can also be obtained directly from the one and two particle density matrices by coupling-constant averaging according to
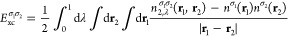
16This can be used to test the numerical convergence of the XC energies calculated from the system- and spherically averaged XC holes with respect to *u*, see eq 13.

At λ = 1, the many-body correlation energy can also be calculated in a similar manner,
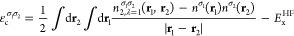
17where *E*_x_^HF^ is the Hartree–Fock exchange energy. Note that the correlation energy defined above is slightly different from the DFT one in that the orbitals used in *E*_x_^HF^ are not the KS orbitals that yield the physical electron density. We therefore use ε_c_ to differentiate from the λ-averaged *E*_c_ of DFT. In this work, ε_c_ is only used for the purpose of testing the basis set convergence of the reference CCSD calculations, see Table 1.

**Table 1:** Basis Set Convergence Analysis for the d-aug-cc-pV*X*Z Basis Sets Using the 2-Point Extrapolation of Reference (42), with *X* = D, T, Q, 5, 6 for He, N and Ne, and *X* = D, T, Q, 5 for Li and Be^a^

atom	basis set	*E*_HF_/*E*_h_	ε_c_^CCSD^/*E*_h_	ε_c_^(T)^/*E*_h_	ε_c_/*E*_h_	*E*_total_/*E*_h_
He	d-aug-cc-pVDZ	–2.85573239	–0.03534795		–0.03534795	–2.89108034
	d-aug-cc-pVTZ	–2.86118442	–0.03995492		–0.03995492	–2.90113933
	d-aug-cc-pVQZ	–2.86152239	–0.04118447		–0.04118447	–2.90270685
	d-aug-cc-pV5Z	–2.86162722	–0.04160561		–0.04160561	–2.90323283
	d-aug-cc-pV6Z	–2.86167323	–0.04179494		–0.04179494	–2.90346816
	Extrap. [56]		–0.04205501		–0.04205501	–2.90372824
	ref (43)	–2.86168			–0.04204	–2.90372
Li	d-aug-cc-pVDZ	–7.43245013	–0.02976686	–0.00002074	–0.02978761	–7.46223773
	d-aug-cc-pVTZ	–7.43270628	–0.03754452	–0.00002408	–0.03756860	–7.47027487
	d-aug-cc-pVQZ	–7.43271947	–0.03979260	–0.00002649	–0.03981909	–7.47253856
	d-aug-cc-pV5Z	–7.43274670	–0.04066065	–0.00003020	–0.04069085	–7.47343755
	Extrap. [Q5]		–0.04157139	–0.00003409	–0.04160548	–7.47435218
	ref (43)	–7.432730			–0.045330	–7.478057
Be	d-aug-cc-pVDZ	–14.57238670	–0.07898150	–0.00036407	–0.07934557	–14.65173227
	d-aug-cc-pVTZ	–14.57287634	–0.08570531	–0.00043926	–0.08614457	–14.65902091
	d-aug-cc-pVQZ	–14.57296957	–0.08779773	–0.00055479	–0.08835252	–14.66132209
	d-aug-cc-pV5Z	–14.57301266	–0.08905158	–0.00058869	–0.08964027	–14.66265294
	Extrap. [Q5]		–0.09036710	–0.00062425	–0.09099135	–14.66400401
	ref (43)	–14.57302			–0.09434	–14.66736
N	d-aug-cc-pVDZ	–54.39339243	–0.13885654	–0.00167501	–0.14053155	–54.53392399
	d-aug-cc-pVTZ	–54.40122030	–0.16133350	–0.00284838	–0.16418188	–54.56540218
	d-aug-cc-pVQZ	–54.40383973	–0.16866385	–0.00316775	–0.17183160	–54.57567133
	d-aug-cc-pV5Z	–54.40447066	–0.17247573	–0.00329317	–0.17576890	–54.58023957
	d-aug-cc-pV6Z	–54.40453785	–0.17427399	–0.00334435	–0.17761834	–54.58215619
	Extrap. [56]		–0.17674413	–0.00341465	–0.18015878	–54.58469663
	ref (43)	–54.40093			–0.18831	–54.58924
Ne	d-aug-cc-pVDZ	–128.49653420	–0.27867301	–0.00412515	–0.28279816	–128.77933230
	d-aug-cc-pVTZ	–128.53330100	–0.33258150	–0.00579453	–0.33837603	–128.87167700
	d-aug-cc-pVQZ	–128.54376810	–0.35601024	–0.00631658	–0.36232682	–128.90609490
	d-aug-cc-pV5Z	–128.54678850	–0.36700350	–0.00657293	–0.37357643	–128.92036490
	d-aug-cc-pV6Z	–128.54706268	–0.37221175	–0.00668601	–0.37889776	–128.92596043
	Extrap. [56]		–0.37936594	–0.00684134	–0.38620728	–128.93326996
	ref (43)	–128.54709			–0.39047	–128.93756

a The CCSD correlation energy is denoted as ε_c_^CCSD^, while ε_c_^(T)^ represents the perturbative triples excitation (T) contribution to the correlation energy, the total correlation energy being given by ε_c_. We use ε_c_ to differentiate from the *λ*-averaged *E*_c_ of DFT. The reference Hartree–Fock energies *E*_HF_ and CAS MCHF correlation energies are taken from ref (43).

## Computational Details

The computational details of the present work are similar to those outlined in ref (26).; the Lieb optimization method implemented in the QUEST(35) program was employed with a CCSD reference wave function and the Gaussian basis expansion approach of Wu and Yang^36^ was used to optimize the potential, *v*_λ_. In this study we use a spin-unrestricted formalism for all calculations to extend the analysis to open-shell electronic states. Dunning basis sets were used in all calculations, specifically the d-aug-cc-pVQZ basis set to represent the orbitals and the aug-cc-pVQZ basis set for the potential, using the uncontracted spherical Gaussian form of these basis sets in all cases.^37−39^ The spherically averaged XC holes of eq 9 were constructed by angular integration using an order-41 Lebedev quadrature grid at each reference point.^40,41^ The PBE and LDA XC model holes^11−13^ were calculated with a *u* interval of 0.01 bohr. Data for the reference and model holes calculated in this work is provided in the Supporting Information.

## Results and Discussion

### Basis Set Extrapolation

To confirm that the basis sets used are sufficiently complete, we examine the basis-set convergence of the coupled-cluster energies, using the d-aug-cc-pV*X*Z series of Dunning basis sets^37−39^ to determine an estimate of the complete basis set (CBS) limit using the 2-point extrapolation of ref (42).
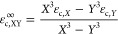
18where *X* and *Y* represent the cardinal numbers in the basis sets used in the extrapolation. Following the recommendation of Halkier et al.,^44^ we do not extrapolate the Hartree–Fock energy, but rather use the energy calculated directly in basis sets with cardinal numbers of 5 or higher. The extrapolated estimates of the CBS energies are therefore obtained by applying eq 18 to the correlation energy and adding this value to the Hartree–Fock energy in the largest basis set available for each system.

The Hartree–Fock, CCSD and perturbative triple excitation (T) correlation energies for this series of basis sets are given in Table 1, alongside the CBS extrapolated values for each of the five atoms. The cardinal numbers used in the extrapolation are indicated in square brackets. Note these calculations are conducted for the physical systems with λ = 1, and thus do not involve the Lieb optimization. The d-aug-cc-pVQZ basis set recovers more than 99% of the CBS value for the total energy for all atoms, and between 93.8% (Ne) and 97.9% (He) of the CBS value for the correlation energy. In addition, the (T) contribution to the total CCSD(T) correlation energy is below 2% at the CBS limit for all atoms considered here. From these results, we consider the CCSD wave function in the d-aug-cc-pVQZ basis set to provide adequate accuracy to be used in the Lieb optimization to compute reference XC holes for these atoms. Furthermore, our CCSD(T) CBS energies agree reasonably well with the Complete Active Space Multi-Configurational Hartree–Fock (CAS MCHF) reference values of ref (43). We note the largest percentage error in the CBS correlation energy compared to the MCHF reference value is observed for lithium, due to its highly diffuse charge density. This discrepancy arises from the limitations of Gaussian functions in accurately representing the diffuse orbitals of lithium, unlike the Slater-type functions used in the MCHF reference, which exhibit the correct attenuation behavior. The small differences in the correlation energies would lead to the CCSD correlation holes that are slightly shallower than the exact correlation holes.

### Convergence Analysis

An essential measure for evaluating the accuracy and reliability of computational methods used to calculate the λ-integrated XC hole is its convergence with respect to the upper limit on *u*, both in recovering the XC energy via eq 13 and in satisfying the sum rule constraints of eq 15. To quantify the XC energy convergence with respect to the upper limit on *u*, we consider the difference between the value of the XC energy given by eq 13, integrated up to some value of *u*, and that evaluated from the CCSD electron density divided by the electron number N, denoted as . For the XC hole shape convergence, we consider the difference between the integral of the exchange/correlation holes up to some value of *u* and the theoretical sum rule given by eq 15; the differences between these are given by |∫_0_^*u*^d*u*′4π*u*′^2^ ⟨*n*_x_^method^(*u*′)⟩ + 1| and |∫_0_^*u*^ d*u*′4π*u*′^2^⟨ *n̅*_c_^method^(*u*′)⟩| for the exchange and correlation holes respectively.

The reference for the convergence behavior for both the XC hole shape and XC hole energy is provided by the Lieb + CCSD λ-integrated system- and spherically averaged holes. Throughout the convergence testing process, we maintain a fixed interval of 0.01 bohr for *u*, while exploring the range of 0 to 15 bohr for Li atom and 0 to 10 bohr for the other atoms with the Lieb + CCSD method and the range from 0 to 100 bohr for testing the model exchange/correlation holes. This allows us to gain a complete picture of the convergence properties of these quantities while avoiding unnecessary computational expense in constructing the Lieb + CCSD reference values over large values of *u* beyond those at which the integrals have converged.

#### Exchange Hole Convergence

**Figure 1:**
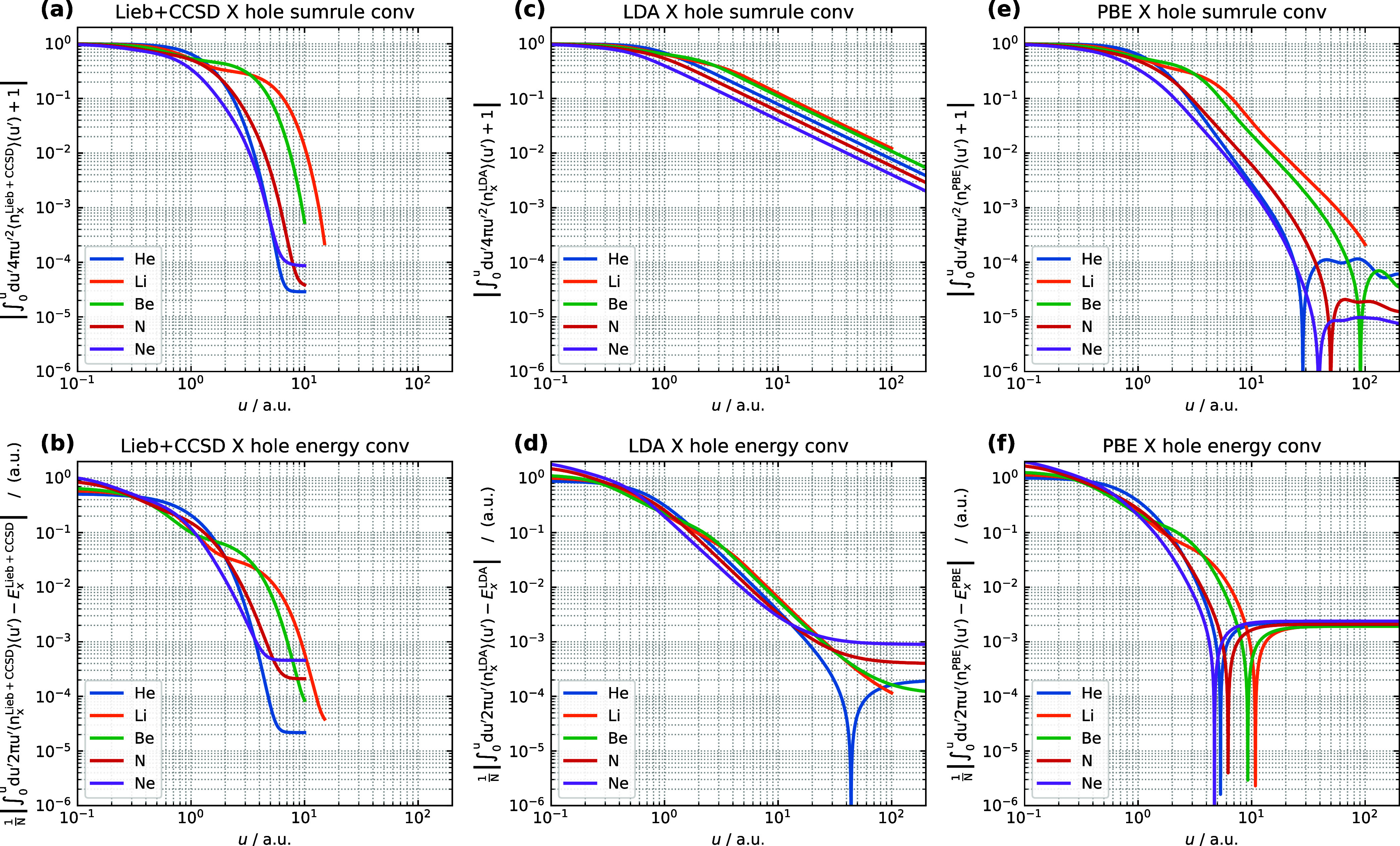
(a, c, e) Sum rule error with respect to the upper limit on *u* for the system- and spherically averaged Lieb + CCSD, LDA, and PBE exchange holes. (b, d, f) *E*_x_ convergence with respect to the upper limit on *u* for the system- and spherically averaged Lieb + CCSD, LDA, and PBE exchange holes. The calculations are performed for the He, Li, Be, N, and Ne atoms using the d-aug-cc-pVQZ orbital basis set and the aug-cc-pVQZ potential basis set. Notably, the LDA and PBE exchange hole models utilize the CCSD electron density and electron density gradient as input.

The convergence measures for the energy and sum rule are presented for the LDA and PBE exchange hole models^12^ alongisde the Lieb + CCSD exchange holes for all five atoms in Figure 1.

It can be seen in Figure 1(a) that, for the Lieb + CCSD exchange holes, the sum rule integrals exhibit relatively rapid convergence with increasing *u*, with the sum rule integral error converging to 10^–3^ or below for all atoms at the maximum values of *u* examined here. The convergence is more rapid for the He, N and Ne atoms, with the sum rule error falling below 10% by *u* = 3 bohr, than for the Be and Li atoms, which reach the same level of convergence at *u* = 5 and *u* = 7 bohr respectively. The sum rule convergence error reaches 1% at *u* = 5 bohr for He, N and Ne and at *u* = 8 and *u* = 10 bohr for Be and Li respectively, while the values become *u* = 6, 9 and 12 bohr respectively for the error to fall below 0.1%.

Regarding the convergence of the exchange energy, shown in Figure 1(b) for the Lieb + CCSD exchange holes, the exchange energy per electron is recovered to within 0.1 *E*_h_ for all atoms in the range *u* < 2 bohr. Convergence to within 0.01 *E*_h_ is reached within *u* = 2 bohr for He, N and Ne while for Be and Li this convergence is achieved at *u* = 5 bohr and *u* = 6 bohr respectively. To reach convergence of 0.001 *E*_h_, the range of *u* must be extended to 5, 7 and 9 bohr for these systems respectively.

The convergence of the LDA model exchange holes, shown in Figure 1(c), is much slower than that of the Lieb + CCSD exchange holes. The sum rule integral error declines approximately exponentially, shown by the near-linear decay on the log–log scale of Figure 1(c), with convergence reached of between 5 and 0.4% for these atoms at *u* = 100 bohr. The exchange energy per electron converges to within 0.1 *E*_h_ for all atoms in the range *u* < 3 bohr and to within 0.01 *E*_h_ in the range *u* < 8 bohr. To reach convergence of 0.001 *E*_h_ requires integration up to 30 bohr for He, N and Ne and up to 50 bohr for Be and Li. The convergence of the exchange energy per electron with respect to *u* exhibits a near-linear trend on the log–log axes of Figure 1(d) in the range 1 < *u* < 10 bohr, tailing off to become approximately constant for Be and Li in the range 20 < *u* < 100 bohr. The data for the He atom exhibits a feature not observed in the other atoms; the convergence becomes much tighter and hits a singularity between 40 and 50 bohr, with the error becoming greater again at larger distances. The sharpness of this minimum is accentuated by the use of logarithmic scales, with a similar effect seen in other plots discussed below. The plateau of finite numerical error at large u after the singularity is likely due to the finite size of u interval of the numerical integration, which is also observed in other figures.

For the PBE exchange hole model, the sum rule integral error is shown in Figure 1(e), in which it can be seen that the behavior is somewhere between that of the Lieb + CCSD and LDA exchange holes. Convergence to within 0.1% is reached for the He, N and Ne atoms by *u* = 20 bohr, while the values for Be and Li are 40 and 60 bohr respectively. Notably, for large values of *u* representing long-range behavior, He, Be, N, and Ne exhibit a significant drop in sum rule integral error convergence, followed by an increase in the error at increasing *u*, although convergence remains below 10^–4^. The convergence of the exchange energy per electron evaluated with the PBE model exchange hole, shown in Figure 1(f), resembles that of the Lieb + CCSD reference in the range 1 < *u* < 4 bohr, with sharp drops in the error being observed for all atoms in the range 4 < *u* < 12, with the error then increasing for all atoms with further increases in *u* to reach an asymptotic level of 2 m*E*_h_.

The difference in behavior of the LDA and PBE exchange hole convergence may be rationalized by considering their shape functions, given by eqs 18 and 24 of ref (12), respectively. The LDA exchange hole shape function is constructed such that the resulting model exchange hole satisfies the sum rule over all space, however as *u* → ∞ the LDA exchange hole integrand varies as ∝ – 1/*u*^2^. The resulting long-range behavior of the LDA model exchange hole leads to the slow convergence of the sum rule error. In comparison, the PBE exchange hole shape function varies as ∝ – e^–*f*(*s*)*u*^2^^/*u*^2^ as *u* → ∞, where *f*(*s*) is a function of the reduced density gradient *s*, resulting in increasing attenuation of the exchange hole as *u* increases and more rapid convergence of the sum rule error. The exchange energy converges much more rapidly than the sum rule with *u* in both cases, which may be understood by comparing eq 11 with eq 15; in the former, the exchange hole is multiplied by *u* in the integrand while in the latter it is multiplied by *u*^2^. The increasing attenuation of the PBE exchange hole model implies steep changes with u, in particular for large *s*, and thus requires a finer u grid to converge, resulting in larger numerical errors of 2 m*E*_h_.

Overall, the convergence of the sum rule error is more rapid for the Lieb + CCSD exchange holes than for either the LDA or PBE model exchange holes, while the convergence of the exchange energies is somewhat similar for the Lieb + CCSD and PBE exchange holes but slower for the LDA exchange hole.

#### Correlation Hole Convergence

**Figure 2:**
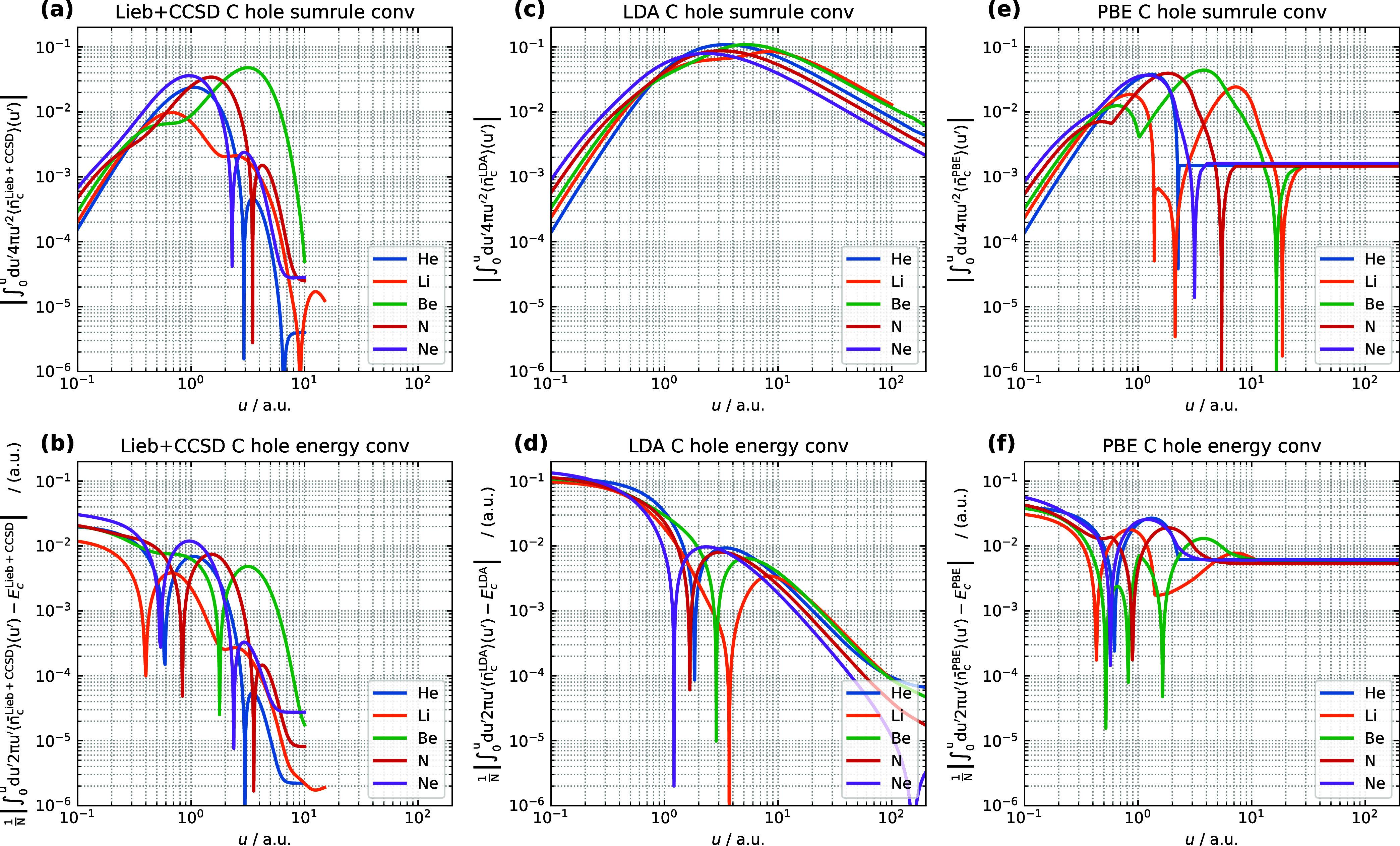
(a, c, e) Sum rule error with respect to the upper limit on *u* for the system- and spherically averaged Lieb + CCSD, LDA, and PBE correlation holes. (b, d, f) *E*_x_ convergence with respect to the upper limit on *u* for the system- and spherically averaged Lieb + CCSD, LDA, and PBE correlation holes. The calculations are performed for the He, Li, Be, N, and Ne atoms using the d-aug-cc-pVQZ orbital basis set and the aug-cc-pVQZ potential basis set. Notably, the LDA and PBE correlation hole models utilize the CCSD electron density and electron density gradient as input.

In an analogous manner to that for the exchange hole, the sum rule and correlation energy convergence for the LDA and PBE model correlation holes are plotted alongside those for the Lieb + CCSD λ-averaged correlation holes for the five atoms in Figure 2.

For the reference Lieb + CCSD correlation hole integrals, shown in Figure 2(a), it can be seen that the value of the integral at *u* = 0 in each case is equal to the on-top correlation hole magnitude, which for these atoms decreases with increasing atomic number: He > Li > Be > *N* > Ne. With increasing *u*, the magnitude of the correlation hole integral then increases for all atoms but reaches a maximum value and begins to decrease again at the first node of the correlation hole. The position at which this occurs is different for each atom, occurring at *u* ∼ 0.6 bohr for Li, *u* ∼ 1–2 bohr for He, N and Ne and *u* ∼ 3 bohr for Be. The value of the integral at this point depends on the depth of the correlation hole up to the first node; this is shallow in Li thus the maximum value of the correlation hole integral is ∼0.01, whereas the greater depth of the correlation hole in the other atoms results in a maximum of ∼0.02–0.05. Beyond this point, the correlation hole becomes positive causing the integral to decrease in magnitude with increasing *u*. Subsequent maxima in the integral may be attributed to the dominance of the positive correlation hole at long-range, amplified by the *u*^2^ prefactor in the integrand, however these are at least an order of magnitude smaller than the initial maxima. There is a general convergence of the correlation hole integral to below 10^–4^ within 10 bohr for all atoms, with the slowest convergence exhibited by Be for which the value of the integral remains above 10^–3^ in the range *u* < 8 bohr.

The convergence of the correlation energy evaluated with the Lieb + CCSD correlation holes are shown in Figure 2(b). In all cases, these exhibit a complicated pattern of convergence as *u* increases. The value at *u* = 0 is simply |*E*_c_^CCSD^| and there is a general trend toward convergence at large *u*, reaching 0.2 m*E*_h_ per electron or below at *u* = 10 bohr. In-between these two values however, the deviation from *E*_c_^CCSD^ for each atom exhibits at least one sharp minimum and one maximum; the positions of the maxima coincide with those in the integral of the correlation hole while the sharp minima indicate the recovery of the correlation energy *E*_c_^CCSD^ of eq 16 from the correlation hole calculations, see eq 13. Since the correlation hole goes above zero as *u* increases, there can be multiple sharp minima for one system, e.g., He, N, and Ne, as shown in Figure 2(b).

The convergence of the LDA correlation hole integral toward the sum rule is shown in Figure 2(c). The values at *u* = 0 are again those of the on-top correlation holes for these atoms, which may be more accurate than those of the Lieb + CCSD reference since the interelectronic cusp is captured in the density functional models.^26^ The correlation hole integrals in the region *u* = 0.1–0.4 bohr follow the trend of those for the Lieb + CCSD reference, reaching a maximum value in the region *u* = 2–7 bohr for all atoms. However, the value of this maximum is greater at around 0.1 and the subsequent decrease in the integral with increasing *u* is much slower than for the Lieb + CCSD reference, being near-linear on the log–log scale of Figure 2(c). This behavior indicates that the LDA model correlation holes are significantly more long-ranged than the reference Lieb + CCSD correlation holes.

Regarding correlation energy convergence, shown in Figure 2(d), the LDA correlation hole model yields significantly higher values at short-range *u* ∼ 0.1 bohr, compared to Lieb + CCSD. Sharp minima in the correlation energy integration error are present around *u* = 2, 4, 3, 2 and 1 bohr for He, Li, Be, N and Ne respectively - indicative of a coincidental agreement of the correlation energy integral, in the region where the correlation hole is negative, and the total correlation energy. As *u* further increases, the error in the integrated correlation energy returns to the previous trend, decreasing near-linearly on the log–log scale in the range *u* ∼ 10–100 bohr, reaching an error in the order of 0.1 m*E*_h_ per electron at *u* = 100 bohr.

The convergence of the PBE model correlation hole to the sum rule is shown in Figure 2(e). The initial values closely resemble those of the LDA correlation hole, with the two models giving a very similar representation of the interelectronic cusp region. The increases in the integrals then somewhat resemble those of the Lieb + CCSD reference, although with apparent discontinuities in the derivatives of the curve for N and Be appearing at around *u* = 0.7 and 1.0 bohr, respectively. The difference in the PBE correlation hole integral and that of the Lieb + CCSD reference for Li is significant beyond around 1.0 bohr, with two sharp decreases between *u* = 1.0–1.1 bohr followed by a large increase to above 0.02 at around *u* ∼ 8 bohr. These features are not present in the Lieb + CCSD reference for Li. A notable difference between the PBE correlation hole integral and that of both LDA and Lieb + CCSD is that the PBE correlation hole integral reaches a value of around 1.5 × 10^–3^ for all atoms with increasing *u*, at which it remains fixed as *u* is increased up to 100 bohr. The discontinuities and the residual at large *u* are likely due to the cutoff step function used in the PBE correlation hole model to enforce the sum rule.^11^ The finite size of *u* interval of the numerical integration is likely not fine enough to accurately capture the step function associated with the cutoff radius *u*_c_.

A similar picture is observed for the convergence of the PBE correlation energy in Figure 2(f). Although the correlation energies are between those of LDA and of Lieb + CCSD and the initial trend at small *u* then follows that seen for the Lieb + CCSD correlation energy convergence, there are a larger number of apparent discontinuities in the derivative of these curves and, by *u* = 10 bohr, the errors in the correlation energy for all atoms converge to around 6 m*E*_h_ beyond which they remain fixed. This is because the PBE correlation hole model is constructed to always satisfy the sum rule, but it does not reproduce the PBE correlation energy functional exactly,^11^ which can be seen in Table 2 by comparing *E*_c_ from the PBE correlation energy functional and those from the PBE correlation hole model.

**Table 2:** Comparison of *E*_xc_ Values Calculated with the LDA and PBE Functionals Evaluated on the CCSD Density Non-Self-Consistently (DFT@CCSD), and Those with the LDA and PBE XC Hole Models Also Evaluated Non-Self-Consistently on the CCSD Density, against Values Obtained by Integrating across the Adiabatic Connection Constructed by Lieb Optimization with a CCSD Reference, Using the d-aug-cc-pVQZ Orbital Basis Set and the aug-cc-pVQZ Potential Basis Set^a^

	non self-consistent DFT@CCSD				XC hole model@CCSD				Lieb + CCSD	
atom	LDA *E*_x_	LDA *E*_c_	PBE *E*_x_	PBE *E*_c_	LDA *E*_x_	LDA *E*_c_	PBE *E*_x_	PBE *E*_c_	*E*_x_	*E*_c_
He	–0.8829	–0.1123	–1.0127	–0.0418	–0.8832	–0.1124	–1.0149	–0.0480	–1.0241	–0.0417
Li	–1.5374	–0.1508	–1.7568	–0.0513	–1.5372	–0.1510	–1.7598	–0.0594	–1.7797	–0.0401
Be	–2.3203	–0.2251	–2.6441	–0.0861	–2.3201	–0.2252	–2.6479	–0.0977	–2.6730	–0.0894
N	–5.8963	–0.4268	–6.5478	–0.1798	–5.8949	–0.4269	–6.5552	–0.1985	–6.5971	–0.1704
Ne	–11.0158	–0.7416	–12.0486	–0.3493	–11.0113	–0.7416	–12.0606	–0.3796	–12.0783	–0.3594

a All energies are given in *E*_h_.

### Coupling-Constant Averaged XC Holes and Hole Models

In the Convergence Analysis section, we analyzed the exchange and correlation holes of LDA, PBE and Lieb + CCSD by examining their convergence properties—key quantitative measures of the accuracy of model exchange and correlation holes. In this Section, we extend this analysis to consider their real-space behavior, providing specific insight into how the model exchange and correlation holes may be improved.

#### Exchange Hole

**Figure 3:**
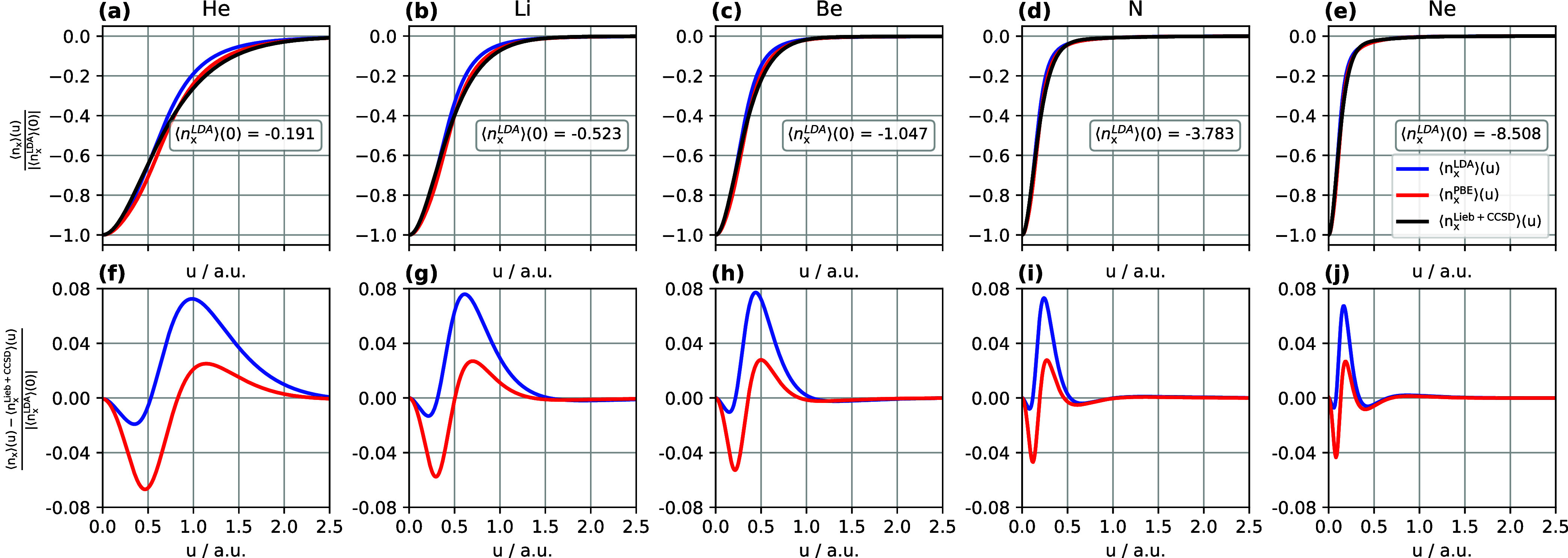
(a–e) Normalized system- and spherically averaged exchange holes computed using Lieb + CCSD, LDA, and PBE. (f–j) Normalized differences between the LDA and PBE exchange holes and the Lieb + CCSD reference. All calculations are performed for the He, Li, Be, N, and Ne atoms using the d-aug-cc-pVQZ orbital basis set and the aug-cc-pVQZ potential basis set. Notably, the LDA and PBE exchange hole models utilize the CCSD electron density and electron density gradient as input.

A normalized system- and spherically averaged exchange hole may be defined as ⟨*n*_x_⟩(*u*)/|⟨*n*_x_^LDA^⟩(0)|, where ⟨*n*_x_^LDA^⟩(0) is the on-top LDA exchange hole, which is expected to be accurate for systems that are well represented by a single determinant.^12^ This normalized hole is a useful quantity with which we can easily assess the similarities and differences between models and is plotted for each atom in the upper panels (a–e) of Figure 3. It can be seen that all three exchange holes are equal at *u* = 0 and that, on this scale, appear to have similar shapes.

The differences of the normalized LDA and PBE exchange holes with respect to the Lieb + CCSD reference are plotted in the lower panels (f–j) of Figure 3, revealing that the normalized LDA exchange hole model exhibits a maximum difference of 0.07–0.08 and, as the nuclear charge increases, the value of *u* at which the greatest positive value of this difference occurs becomes smaller, decreasing from 1.0 bohr in He to 0.2 bohr in Ne, indicating the exchange holes become more localized. The first minimum in these differences for LDA varies from −0.02 to −0.005, approaching zero as the nuclear charge increases. Overall, Figure 3(f–j) show that the LDA exchange holes are shallower than the Lieb + CCSD reference exchange holes.

In contrast, it can be observed from Figure 3(f–j) that the normalized PBE exchange hole model exhibits significantly more negative differences from the reference compared to the normalized LDA exchange hole model, indicating that the PBE exchange hole is deeper than the LDA exchange hole. The negative difference with the largest magnitude is around −0.06 for He, decreasing in magnitude as the nuclear charge increases to around −0.04 for Ne. Meanwhile the largest positive difference remains constant at around 0.02 for each of the five atoms, with the value of *u* at which it occurs decreasing from around 1.1 bohr to 0.2 bohr from He to Ne. Overall, the PBE exchange holes still have considerable deviations from the Lieb + CCSD exchange holes, but these are much more evenly balanced between positive and negative differences, while overall being closer to the Lieb + CCSD reference than those of LDA. Consequently, the PBE exchange hole model outperforms the LDA exchange hole model in terms of the exchange energy, *E*_x_, as demonstrated in Table 2.

#### Correlation Hole

**Figure 4:**
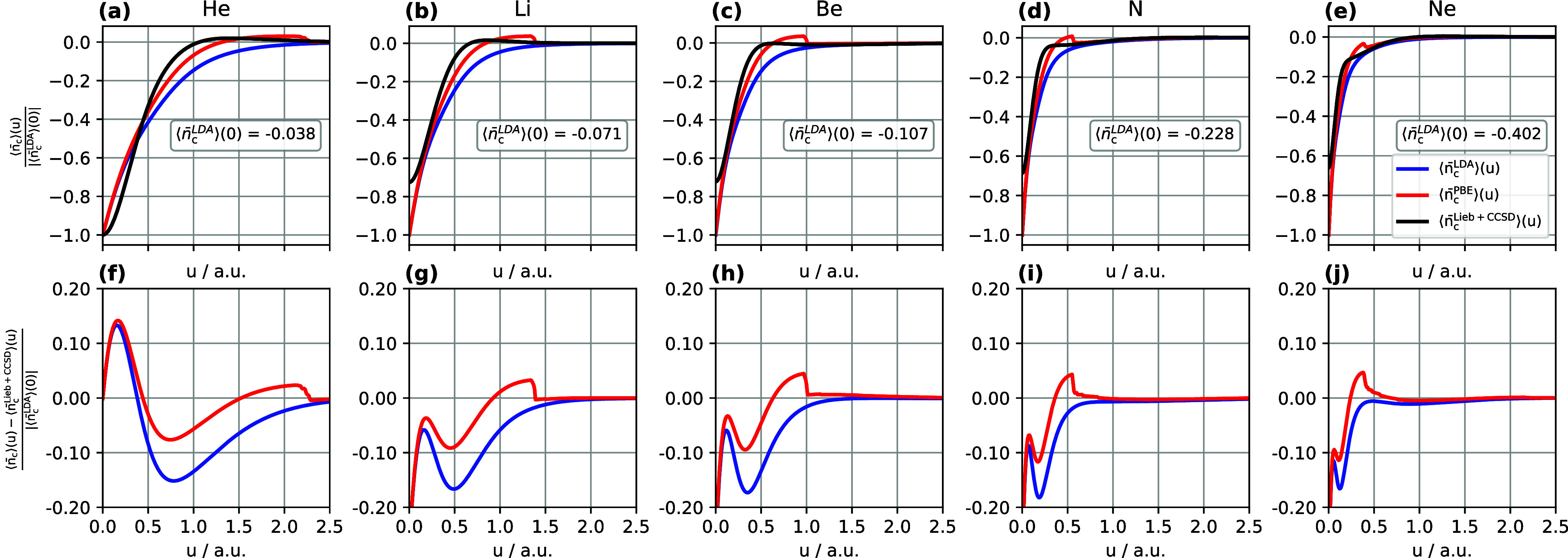
(a–e) Normalized system- and spherically averaged correlation holes computed using Lieb + CCSD, LDA, and PBE. (f–j) Normalized differences between the LDA and PBE correlation holes and the Lieb + CCSD reference. All calculations are performed for the He, Li, Be, N, and Ne atoms using the d-aug-cc-pVQZ orbital basis set and the aug-cc-pVQZ potential basis set. Notably, the LDA and PBE correlation hole models utilize the CCSD electron density and electron density gradient as input.

Similar to the exchange hole, the normalized correlation hole provides a convenient measure for comparing different correlation hole models. It is calculated as ⟨*n̅*_c_⟩(*u*)/|⟨ *n̅*_c_^LDA^⟩(0)|, where the LDA correlation hole model is expected to provide a reasonably accurate description of the on-top correlation hole value ⟨*n̅*_c_⟩(0).^45^ The normalized LDA, PBE and Lieb + CCSD correlation holes are shown in Figure 4(a–e), in which we observe that only for He does the on-top correlation hole of Lieb + CCSD match the value obtained from the DFT correlation hole models. For the other atoms, the normalized Lieb + CCSD on-top correlation hole exhibits deviations of approximately 0.3 from the DFT models. As the nuclear charge increases, these deviations become more pronounced due to the increasing significance of the electron–electron cusp, which is poorly described in finite basis set calculations for CCSD.

The differences in the normalized correlation holes with respect to Lieb + CCSD are shown in Figure 4(f–j), clearly revealing an initial region corresponding to the characteristic radius (identified by the first peak) of the cusp-region errors described in ref (26). Inside this radius the Lieb + CCSD reference for the correlation hole fails to adequately describe the electron–electron cusp, owing to the finite basis set correlated calculations upon which it is based. In contrast, the LDA and PBE models inside this radius agree well with each other and by construction exhibit a cusp. It has been argued that, while not exact, the value of ⟨*n̅*_c_^LDA^⟩(0) may be rather accurate,^45^ suggesting that in this near-cusp region these simple models may even exceed the accuracy of the reference data. Beyond the near-cusp region, the Lieb + CCSD correlation hole can be considered to be more reliable than the DFT models for these systems, as shown in ref (26). In Figure 4(f–j), for values of *u* beyond the near-cusp region, the DFT correlation hole models exhibit predominantly negative differences with respect to the Lieb + CCSD reference, most notably for the LDA correlation hole model. The most negative value of this difference is approximately −0.17, arising from the LDA model correlation hole at *u* = 0.2 in the N atom. The PBE correlation hole model shows negative differences from the reference at similar values of *u* to the LDA correlation hole model, but with magnitudes that are approximately half of those from the LDA models.

With the exception of the He atom near the cusp, the LDA correlation hole model yields negative differences with respect to Lieb + CCSD across the full range of *u* for all atoms, indicating that the LDA correlation hole model is too deep with respect to the reference one. On the other hand, the PBE correlation hole model exhibits positive differences with respect to Lieb + CCSD in all atoms, with the maximum value increasing from 0.02 to 0.05 from He to Ne, and the value of *u* at which this occurs reducing from 2.2 bohr to 0.4 bohr. These observations indicate that the PBE correlation hole model provides a more accurate estimation of *E*_c_ compared to the LDA correlation hole model, as demonstrated in Table 2. Due to the step-function present in the definition of the PBE correlation hole, the normalized PBE correlation hole exhibits a discontinuity at some value of *u* for all atoms, with this value becoming smaller with increasing atomic number.

#### XC Hole

**Figure 5:**
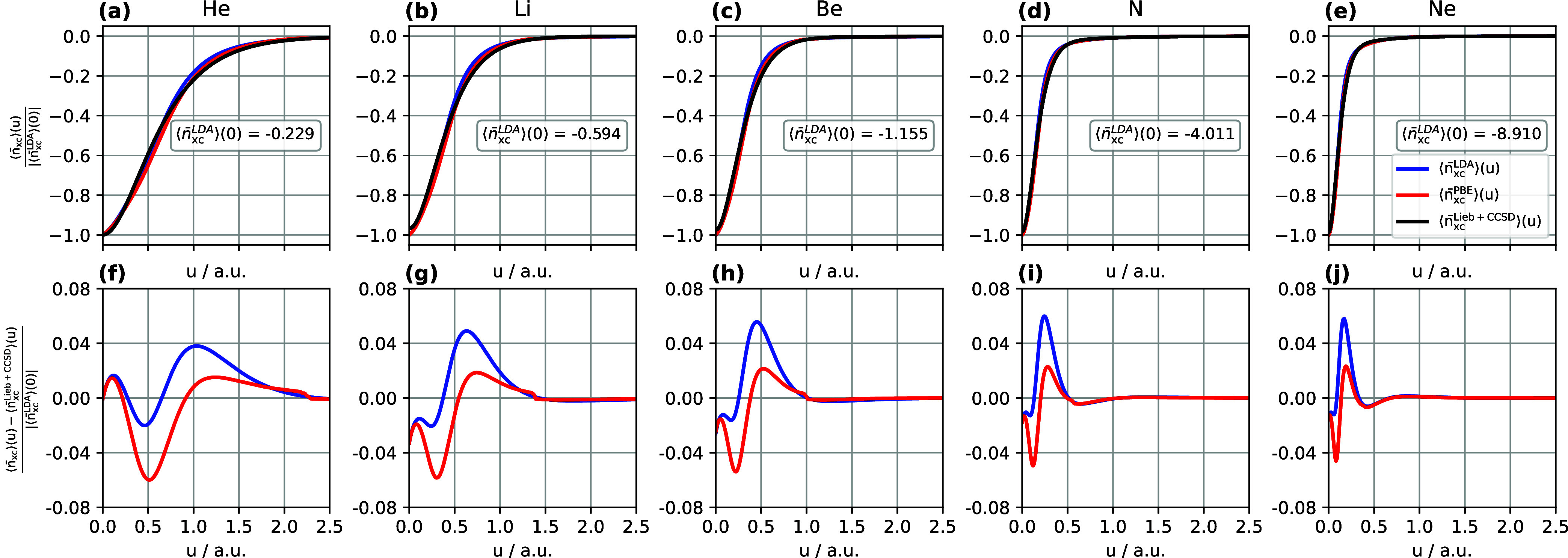
(a–e) Normalized system- and spherically averaged exchange–correlation holes computed using Lieb + CCSD, LDA, and PBE. (f–j) Normalized differences between the LDA and PBE exchange–correlation holes and the Lieb + CCSD reference. All calculations are performed for the He, Li, Be, N, and Ne atoms using the d-aug-cc-pVQZ orbital basis set and the aug-cc-pVQZ potential basis set. Notably, the LDA and PBE exchange–correlation hole models utilize the CCSD electron density and electron density gradient as input.

From the previous discussion analyzing the exchange and correlation holes individually, we can observe a qualitative trend indicating that the PBE XC hole model has smaller errors in *E*_x_ and *E*_c_ separately compared to the LDA XC hole model. Previous analysis also indicates that the LDA exchange holes are shallower than the Lieb + CCSD reference exchange holes, while the LDA correlation holes are deeper than the corresponding reference ones. Therefore, smaller deviations of the LDA XC holes from the reference Lieb + CCSD XC holes are observed in Figure 5(f–j), illustrated by the smaller peaks of around 0.04 in comparison with those of around 0.08 given in Figure 3(f–j). This demonstrates the error cancellation between the LDA exchange and correlation contributions. Similar error cancellations are observed for the PBE exchange and correlation hole models as shown in Figures 3–5, but to a lesser extent since each component individually provides a closer representation of the Lieb + CCSD exchange and correlation holes than those of the LDA models.

## Conclusions

In this work, we have presented the system- and spherically averaged XC holes for the helium, lithium, beryllium, nitrogen and neon atoms at the CCSD level of accuracy. These quantities were constructed along the adiabatic connection using the Lieb optimization method for varying coupling strengths, λ, and then coupling strength averaged to provide accurate benchmarks appropriate for comparison with DFT XC hole models. In addition, this approach yields accurate values of *E*_x_ and *E*_c_ against which those evaluated with DFT models may be compared.

A thorough numerical analysis of the Lieb + CCSD exchange and correlation holes was carried out, confirming their adherence to the sum rules for exchange and correlation holes and the recovery of the exchange and correlation energies when integrated with respect to the interelectronic separation *u*. These benchmark quantities were also compared with LDA and PBE model exchange and correlation holes. While the Lieb + CCSD exchange holes demonstrated rapid convergence with respect to *u* both in respect of the sum rule and exchange energy, the LDA and PBE exchange hole models show a more gradual convergence with increasing *u*. Examination of the shape functions for the LDA and PBE model exchange holes provided a rationalization for the improved convergence properties of the PBE model exchange hole in comparison with the LDA model.

Different trends were observed for the convergence behavior of the correlation holes compared with that for the exchange holes. The convergence of the LDA correlation hole sum rule exhibits a near-linear trend on the log–log scale as *u* becomes large, deviating significantly from the Lieb + CCSD reference. The PBE correlation hole sum rule is more similar to the Lieb + CCSD reference at low *u* but features many apparent derivative discontinuities with increasing *u* and tends to a constant value for all atoms at large *u*. These undesirable features were attributed to the presence of a step-function in the PBE correlation hole model. The convergence of the correlation energy highlighted the need to consider a relatively large range of *u* values to adequately sample both negative and positive regions of the correlation holes.

We concluded our analysis with an examination of the real-space characteristics of the normalized LDA and PBE exchange and correlation holes, in comparison to those of the Lieb + CCSD reference. We observed that both the exchange and correlation holes become more localized as the nuclear charge increases. The exchange hole shape remained similar for all of the atoms, while the first maximum in the correlation hole decreased in magnitude as the nuclear charge increased. Furthermore, we found that there is generally a greater cancellation of errors between the LDA exchange and correlation hole models than for PBE. Interestingly, it was noted that the short-range (small *u*) cusp in the correlation hole is not well described by the finite basis set Lieb + CCSD calculations, and that in this region the simple LDA and PBE models may be more accurate.

In summary, our analysis provides insight into the characteristics and performance of simple exchange, correlation and combined XC hole models. In future work we aim to utilize the understanding of the strengths and weaknesses of these simple model XC holes to develop significantly improved models to underpin the development of improved density-functional approximations.

## Supporting Information Available

Data for the benchmark and model exchange–correlation holes (ZIP)

## References

[ref1] Hohenberg P.; Kohn W. Inhomogeneous Electron Gas. Phys. Rev. 1964, 136, B864–B871. 10.1103/PhysRev.136.B864.

[ref2] Kohn W.; Sham L. J. Self-Consistent Equations Including Exchange and Correlation Effects. Phys. Rev. 1965, 140, A1133–A1138. 10.1103/PhysRev.140.A1133.

[ref3] Parr R.; Yang W. Density-Functional Theory of Atoms and Molecules; Oxford University Press: New York, 1989.

[ref4] Gunnarsson O.; Lundqvist B. I. Exchange and correlation in atoms, molecules, and solids by the spin-density-functional formalism. Phys. Rev. B 1976, 13, 4274 10.1103/PhysRevB.13.4274.

[ref5] Constantin L. A.; Fabiano E.; Sala F. D. Construction of a general semilocal exchange-correlation hole model: Application to nonempirical meta-GGA functionals. Phys. Rev. B 2013, 88, 125112 10.1103/PhysRevB.88.125112.

[ref6] Langreth D.; Perdew J. The exchange-correlation energy of a metallic surface. Solid State Commun. 1975, 17, 1425–1429. 10.1016/0038-1098(75)90618-3.

[ref7] Perdew J. P.; Kurth S. A Primer in Density Functional Theory; Fiolhais C.; Nogueira F.; Marques M., Eds.; Springer: Berlin Heidelberg, 2003; pp 1–55.

[ref8] McCarty R. J.; Perchak D.; Pederson R.; Evans R.; Qiu Y.; White S. R.; Burke K. Bypassing the Energy Functional in Density Functional Theory: Direct Calculation of Electronic Energies from Conditional Probability Densities. Phys. Rev. Lett. 2020, 125, 266401 10.1103/PhysRevLett.125.266401. 33449722

[ref9] Perdew J. P.; Chevary J. A.; Vosko S. H.; Jackson K. A.; Pederson M. R.; Singh D. J.; Fiolhais C. Atoms, molecules, solids, and surfaces: Applications of the generalized gradient approximation for exchange and correlation. Phys. Rev. B 1992, 46, 6671–6687. 10.1103/PhysRevB.46.6671. 10002368

[ref10] Perdew J. P.; Burke K.; Ernzerhof M. Generalized gradient approximation made simple. Phys. Rev. Lett. 1996, 77, 3865 10.1103/PhysRevLett.77.3865. 10062328

[ref11] Perdew J. P.; Burke K.; Wang Y. Generalized gradient approximation for the exchange-correlation hole of a many-electron system. Phys. Rev. B 1996, 54, 16533–16539. 10.1103/PhysRevB.54.16533. 9985776

[ref12] Ernzerhof M.; Perdew J. P. Generalized gradient approximation to the angle- and system-averaged exchange hole. J. Chem. Phys. 1998, 109, 3313–3320. 10.1063/1.476928.

[ref13] Perdew J. P.; Wang Y. Pair-distribution function and its coupling-constant average for the spin-polarized electron gas. Phys. Rev. B 1992, 46, 12947 10.1103/PhysRevB.46.12947. 10003333

[ref14] Vydrov O. A.; Scuseria G. E. Assessment of a long-range corrected hybrid functional. J. Chem. Phys. 2006, 125, 234109 10.1063/1.2409292. 17190549

[ref15] Tao J.; Perdew J. P.; Staroverov V. N.; Scuseria G. E. Climbing the density functional ladder: Nonempirical meta-generalized gradient approximation designed for molecules and solids. Phys. Rev. Lett. 2003, 91, 146401 10.1103/PhysRevLett.91.146401. 14611541

[ref16] Constantin L. A.; Perdew J. P.; Tao J. Meta-generalized gradient approximation for the exchange-correlation hole with an application to the jellium surface energy. Phys. Rev. B 2006, 73, 205104 10.1103/PhysRevB.73.205104.

[ref17] Sun J.; Ruzsinszky A.; Perdew J. P. Strongly Constrained and Appropriately Normed Semilocal Density Functional. Phys. Rev. Lett. 2015, 115, 036402 10.1103/PhysRevLett.115.036402. 26230809

[ref18] Vydrov O. A.; Van Voorhis T. Nonlocal van der Waals density functional made simple. Phys. Rev. Lett. 2009, 103, 063004 10.1103/PhysRevLett.103.063004. 19792562

[ref19] Constantin L. A.; Pitarke J. M. The many-body exchange-correlation hole at metal surfaces. J. Chem. Theory Comput. 2009, 5, 895–901. 10.1021/ct800553t. 26609598

[ref20] O’Neill D. P.; Gill P. M. W. Wave functions and two-electron probability distributions of the Hooke’s-law atom and helium. Phys. Rev. A 2003, 68, 022505 10.1103/PhysRevA.68.022505.

[ref21] Cioslowski J.; Liu G. Electron intracule densities and Coulomb holes from energy-derivative two-electron reduced density matrices. J. Chem. Phys. 1998, 109, 8225–8231. 10.1063/1.477484.

[ref22] Lieb E. H. Density functionals for coulomb systems. Int. J. Quantum Chem. 1983, 24, 243–277. 10.1002/qua.560240302.

[ref23] Wu Q.; Yang W. A direct optimization method for calculating density functionals and exchange–correlation potentials from electron densities. J. Chem. Phys. 2003, 118, 2498–2509. 10.1063/1.1535422.

[ref24] Teale A. M.; Coriani S.; Helgaker T. The calculation of adiabatic-connection curves from full configuration-interaction densities: Two-electron systems. J. Chem. Phys. 2009, 130, 104111 10.1063/1.3082285. 19292527

[ref25] Teale A. M.; Coriani S.; Helgaker T. Accurate calculation and modeling of the adiabatic connection in density functional theory. J. Chem. Phys. 2010, 132, 164115 10.1063/1.3380834. 20441266

[ref26] Hou L.; Irons T. J.; Wang Y.; Furness J. W.; Wibowo-Teale A. M.; Sun J. Capturing the electron-electron cusp with the coupling-constant averaged exchange-correlation hole: A case study for Hooke’s atoms. J. Chem. Phys. 2024, 160, 014103 10.1063/5.0173370. 38180252

[ref27] Kalita B.; Li L.; McCarty R. J.; Burke K. Learning to Approximate Density Functionals. Acc. Chem. Res. 2021, 54, 818–826. 10.1021/acs.accounts.0c00742. 33534553

[ref28] Fiedler L.; Shah K.; Bussmann M.; Cangi A. Deep dive into machine learning density functional theory for materials science and chemistry. Phys. Rev. Mater. 2022, 6, 040301 10.1103/PhysRevMaterials.6.040301.

[ref29] Wu J.; Pun S.-M.; Zheng X.; Chen G. Construct exchange-correlation functional via machine learning. J. Chem. Phys. 2023, 159, 090901 10.1063/5.0150587. 37671956

[ref30] Kirkpatrick J.; McMorrow B.; Turban D. H. P.; et al. Pushing the frontiers of density functionals by solving the fractional electron problem. Science 2021, 374, 1385–1389. 10.1126/science.abj6511. 34882476

[ref31] Zhao H.; Gould T.; Vuckovic S. Deep Mind 21 functional does not extrapolate to transition metal chemistry. Phys. Chem. Chem. Phys. 2024, 26, 12289–12298. 10.1039/D4CP00878B. 38597718 PMC11041869

[ref32] Cuierrier E.; Roy P.-O.; Ernzerhof M. Constructing and representing exchange–correlation holes through artificial neural networks. J Chem Phys 2021, 155, 174121 10.1063/5.0062940. 34742211

[ref33] Davidson E. R. Reduced Density Matrices in Quantum Chemistry; Academic Press: New York, 1976.

[ref34] McWeeny R. Some Recent Advances in Density Matrix Theory. Rev. Mod. Phys. 1960, 32, 335–369. 10.1103/RevModPhys.32.335.

[ref35] QUEST, A rapid development platform for QUantum Electronic Structure Techniques 2024. https://quest.codes/.

[ref36] Wu Q.; Yang W. Algebraic equation and iterative optimization for the optimized effective potential in density functional theory. J. Theor. Comput. Chem. 2003, 02, 627–638. 10.1142/S0219633603000690.

[ref37] Dunning T. H. Gaussian Basis Sets for Use in Correlated Molecular Calculations. I. The Atoms Boron through Neon and Hydrogen. J. Chem. Phys. 1989, 90, 1007–1023. 10.1063/1.456153.

[ref38] Woon D. E.; Dunning T. H. Gaussian Basis Sets for Use in Correlated Molecular Calculations. III. The Atoms Aluminum through Argon. J. Chem. Phys. 1993, 98, 1358–1371. 10.1063/1.464303.

[ref39] Woon D. E.; Dunning T. H. Gaussian Basis Sets for Use in Correlated Molecular Calculations. V. Core-valence Basis Sets for Boron through Neon. J. Chem. Phys. 1995, 103, 4572–4585. 10.1063/1.470645.

[ref40] Lebedev V. Quadratures on a sphere. USSR Comput. Math. Math. Phys. 1976, 16, 10–24. 10.1016/0041-5553(76)90100-2.

[ref41] Lebedev V. I.; Skorokhodov A. L. Quadrature formulas for a sphere of orders 41, 47 and 53. Dokl. Akad. Nauk 1992, 324, 519–524.

[ref42] Halkier A.; Helgaker T.; Jørgensen P.; Klopper W.; Koch H.; Olsen J.; Wilson A. K. Basis-set convergence in correlated calculations on Ne, N2, and H2O. Chem. Phys. Lett. 1998, 286, 243–252. 10.1016/S0009-2614(98)00111-0.

[ref43] Davidson E. R.; Hagstrom S. A.; Chakravorty S. J.; Umar V. M.; Fischer C. F. Ground-state correlation energies for two-to ten-electron atomic ions. Phys. Rev. A 1991, 44, 7071 10.1103/PhysRevA.44.7071. 9905848

[ref44] Halkier A.; Helgaker T.; Jørgensen P.; Klopper W.; Olsen J. Basis-set convergence of the energy in molecular Hartree–Fock calculations. Chem. Phys. Lett. 1999, 302, 437–446. 10.1016/S0009-2614(99)00179-7.

[ref45] Burke K.; Perdew J. P.; Langreth D. C. Is the local density approximation exact for short wavelength fluctuations?. Phys. Rev. Lett. 1994, 73, 1283 10.1103/PhysRevLett.73.1283. 10057671

